# Minor Histocompatibility Antigen-Specific T Cells

**DOI:** 10.3389/fped.2020.00284

**Published:** 2020-06-03

**Authors:** Corinne Summers, Vipul S. Sheth, Marie Bleakley

**Affiliations:** ^1^Clinical Research Division, Fred Hutchinson Cancer Research Center, Seattle, WA, United States; ^2^Department of Pediatrics, University of Washington, Seattle, WA, United States

**Keywords:** minor histocompatibility antigen, T cell immunotherapy, hematopoietic stem cell transplantation, leukemia, pediatric, graft-vs.-leukemia, graft engineering, polymorphism

## Abstract

Minor Histocompatibility (H) antigens are major histocompatibility complex (MHC)/Human Leukocyte Antigen (HLA)-bound peptides that differ between allogeneic hematopoietic stem cell transplantation (HCT) recipients and their donors as a result of genetic polymorphisms. Some minor H antigens can be used as therapeutic T cell targets to augment the graft-vs.-leukemia (GVL) effect in order to prevent or manage leukemia relapse after HCT. Graft engineering and post-HCT immunotherapies are being developed to optimize delivery of T cells specific for selected minor H antigens. These strategies have the potential to reduce relapse risk and thereby permit implementation of HCT approaches that are associated with less toxicity and fewer late effects, which is particularly important in the growing and developing pediatric patient. Most minor H antigens are expressed ubiquitously, including on epithelial tissues, and can be recognized by donor T cells following HCT, leading to graft-vs.-host disease (GVHD) as well as GVL. However, those minor H antigens that are expressed predominantly on hematopoietic cells can be targeted for selective GVL. Once full donor hematopoietic chimerism is achieved after HCT, hematopoietic-restricted minor H antigens are present only on residual recipient malignant hematopoietic cells, and these minor H antigens serve as tumor-specific antigens for donor T cells. Minor H antigen-specific T cells that are delivered as part of the donor hematopoietic stem cell graft at the time of HCT contribute to relapse prevention. However, in some cases the minor H antigen-specific T cells delivered with the graft may be quantitatively insufficient or become functionally impaired over time, leading to leukemia relapse. Following HCT, adoptive T cell immunotherapy can be used to treat or prevent relapse by delivering large numbers of donor T cells targeting hematopoietic-restricted minor H antigens. In this review, we discuss minor H antigens as T cell targets for augmenting the GVL effect in engineered HCT grafts and for post-HCT immunotherapy. We will highlight the importance of these developments for pediatric HCT.

## Introduction

Allogeneic hematopoietic stem cell transplantation (HCT) is widely employed in the management of very high-risk leukemia in children, adolescents, and young adults (1, 2), because HCT as consolidation therapy is generally associated with a reduced relapse risk compared to chemotherapy alone (3, 4). Although HCT reduces the risk, relapse remains the major cause of death following HCT for leukemia (5). Reported post-HCT relapse rates are variable, with rates of 10–30% for patients transplanted with leukemia in minimal residual disease (MRD) negative remission, 20–70% for those in remission but with MRD, and 50–90% for those in relapse (6, 7). Long-term survival following relapse after HCT is infrequent (8–10).

HCT involves two important elements that confer protection from relapse: first, “conditioning” incorporating chemotherapy and/or radiotherapy as the pre-HCT preparative regimen, and second, donor lymphocytes in the hematopoietic cell graft as cellular therapy. Donor T cells respond to non-donor antigens on recipient cells, including minor histocompatibility (H) antigens, and thereby lead to a graft-vs.-leukemia (GVL) effect when those antigens are presented on leukemic cells. Minor Histocompatibility (H) antigens are Human Leukocyte Antigen (HLA)-presented peptides derived from normal self-proteins that differ in amino acid sequence between donor and recipient due to genetic polymorphisms outside of the chromosome 6 HLA loci (11). The GVL effect and intensive conditioning both substantially contribute to relapse prevention in myeloablative HCT, while the GVL effect is particularly critical with use of less intensive, “non-myeloablative” and “reduced intensity” HCT preparative regimens.

The efficacy of HCT for pediatric leukemia currently depends highly on both the GVL effect and on delivery of myeloablative chemo(radio)therapy in the pre-HCT conditioning regimen. Intense myeloablative conditioning, particularly involving total body irradiation (TBI), causes serious short- and long-term adverse effects, including growth and neurocognitive impairments in pediatric patients (12–16). Thus, there is a critical need to advance HCT strategies that allow reduced conditioning intensity and associated toxicity, while mitigating relapse risk. Furthermore, avoiding severe and chronic graft-vs.-host disease (GVHD) in children is highly desirable due to the morbidity, mortality, disability, and social handicaps, and late effects associated with chronic GVHD (12, 15, 17).

In this review, we discuss minor H antigens as T cell targets for augmenting the GVL effect in engineered HCT grafts and for post-HCT immunotherapy. We will highlight the importance of these developments for pediatric HCT.

## Minor Histocompatibility Antigens

**Figure 1:**
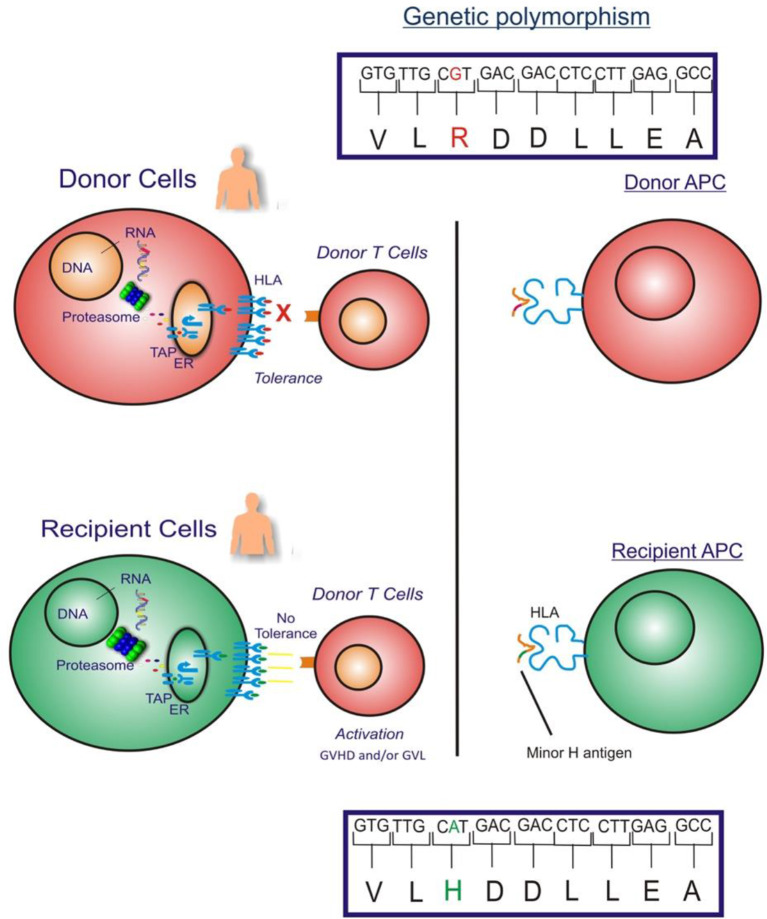
The figure illustrates the GVHD and/or GVL effect in allogeneic HLA-matched donor HCT which is largely attributed to donor T cell responses to recipient cell minor H antigens. When an HCT recipient has a homozygous or heterozygous polymorphism that encodes a minor H antigen and their donor is homozygous negative for the allele, the donor may have T cells in their repertoire that can target the minor H antigen peptide/HLA complex on the recipient's leukemia cell surface leading to an elimination of any residual leukemia following HCT.

The GVL effect in allogeneic HLA-matched related or unrelated donor HCT is largely attributed to T cell responses to recipient minor H antigens. Specifically, when a HCT recipient has a homozygous or heterozygous polymorphism that encodes a minor H antigen and their HLA-matched donor is homozygous for the “negative” allele, the donor may have T cells in their repertoire that recognize the minor H antigen peptide/HLA complex on the recipient's leukemia cell surface, and those cells may eliminate any residual leukemia after HCT (Figure 1).

From the perspective of the donor T cells, minor H antigens are foreign antigens and consequently donor T cells specific for minor H antigens are not subject to self-tolerance mechanisms, allowing for highly avid minor H antigen-specific T cell responses. Most known minor H antigens arise from single nucleotide substitutions in the coding sequences of homologous donor and recipient genes, which change peptide-HLA binding or T cell receptor (TCR) recognition of the peptide-HLA complex. There are at least 660,000,000 single nucleotide polymorphisms (SNPs) and insertion-deletion polymorphisms (indels) in the human genome (18) although less than 1% of SNPs are non-synonymous limiting the number of potential minor H antigens (19). Moreover, only non-synonymous SNPs that give rise to recipient donor mismatches in the graft-vs.-host direction (recipient homozygous positive or heterozygous, donor homozygous negative for the immunogenic allelic variant) are relevant to the GVL effect. T cell recognition of most minor H antigens is unidirectional, mostly due to the lack of T cell recognition of the allelic variant peptide despite cell surface presentation with HLA molecules (20). Alternatively, the corresponding donor peptide may not be generated (21–25), transported by antigen processing machinery (26), escape proteasomal degradation (27), or stably bind to MHC molecules (28–31).

## Minor H Antigens and Selective GVL

HCT outcome data demonstrate the potency of the GVL effect, with reduced relapse rates noted in patient cohorts that develop acute and/or chronic GVHD following allogeneic HCT (32–34). However, the GVL effect is apparently separable from GVHD; reduced relapse rates are still observed in patients who underwent allogeneic HCT and did not develop clinically significant GVHD, as compared to relapse rates in syngeneic HCT recipients (33).

**Figure 2:**
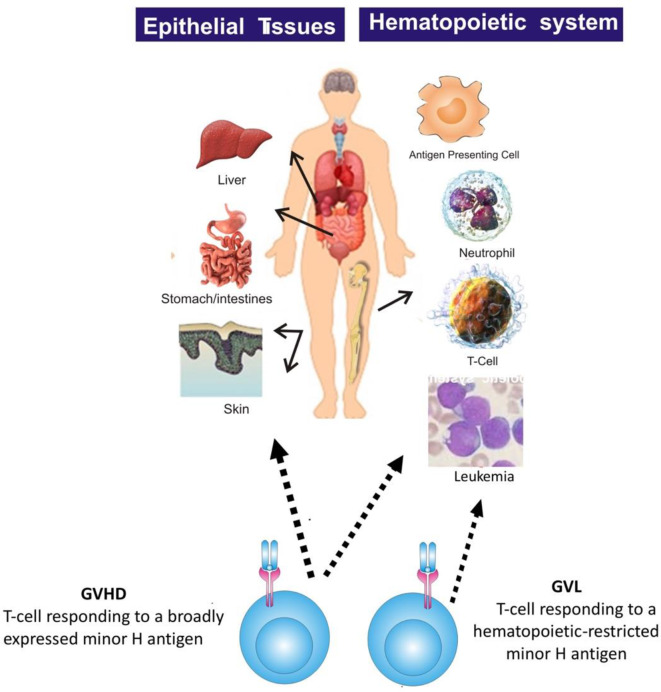
The figure illustrates that most minor H antigens are ubiquitously expressed on epithelial tissues and hematopoietic cells. Donor T cell recognition of such antigens leads to GVHD as well as GVL. However, minor H antigens expressed predominantly on hematopoietic cells can be targets of a selective GVL effect.

**Figure 3:**
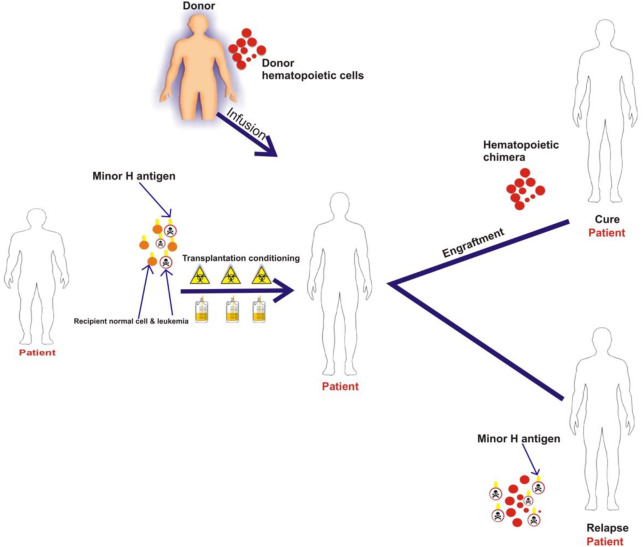
The schematic illustrates that patient normal hematopoietic and leukemia cells with minor H antigens are eliminated by the transplant conditioning regimen and replaced with donor hematopoietic cells. Once full donor normal hematopoietic cell chimerism is achieved only recipient malignant hematopoietic cells present hematopoietic-restricted minor H antigens if disease persists or recurs following HCT. These minor H antigens serve as tumor-specific antigens for donor T cells.

Most minor H antigens are expressed ubiquitously, including on epithelial tissues. Recognition of such minor H antigens by donor T cells following HCT can potentially lead to GVHD as well as GVL (35, 36). However, those minor H antigens that are expressed predominantly on hematopoietic cells can be targets of a selective GVL effect (37, 38) (Figure 2). Once full donor normal hematopoietic chimerism is achieved after HCT, only residual or recurrent recipient malignant hematopoietic cells will present hematopoietic-restricted minor H antigens, and these minor H antigens serve as tumor-specific antigens for donor T cells (Figure 3). Over 100 fully-characterized or candidate human minor H antigens have been identified (22, 25, 28, 39–52). Of these, an important minority are expressed predominantly or exclusively on hematopoietic cells and are of particular interest as targets for GVL-augmenting therapeutic T cells delivered with or following HCT (Table 1). Examples, of well-characterized minor H antigens of high therapeutic interest include HA-1, ACC-1, ACC-2, and LRH1, described below.

**Table 1:** Selected hematopoietic-restricted minor H antigens of interest for therapeutic targeting.

**Minor H antigen (reference)**	**Gene/Chromosome**	**HLA allele**	**Heme/non-heme expression**	**Polymorphism**	**Immunogenic peptide Epitope (underlined)**	**Genotype-phenotype frequencies (%)**	**Estimated disparity MSD (%)**	**Estimated disparity MUD (%)**
HA-1(28, 39)	HMHA1/19p13.3	A*02:01 A*02:06	1–2 logs higher in heme.	rs1801284	VL[H**/R]** DDLLEA	H/H = 13 H/R = 45.8 R/R = 41.2	6.4 +A206 <1	11.6 +A206 <1
LRH-1(22)	P2X5/17p13.3 (frameshift mutation)	B*07:02	1.5–2.0 logs higher in heme	rs3215407	TPNQRQNVC	+/+ = 4 +/– = 50 –/– = 46	4.9	7.5
LB-EBI3-1(40)	EBI3/ 19p13.3	B*07:02	2 logs higher in heme	rs4740	RPRARYY[**I/V]** QV	I/I = 10.6 I/V38.1 V/V = 51.3	3.7	7.5
HB-1(41–43)	HMHB1/ 5q31.3	B*4402 B*4403	B cell	rs161557	EEKRGSL[Y/H] VW	Y/Y = 5.2 H/Y = 41.2 H/H = 53.7	3.9 (Y) 1.2 (H)	6.8 (Y) 1.3 (H)
ACC-2 (44)	BCL2A1/ 15q24.3	B*44:03	1–2 logs higher in heme.	rs3826007	KEFED[D**/G]** IINW	D/D = 6.4 D/G = 38.1 G/G = 55.5	3.6	6.7
ACC-1 (44, 45)	BCL2A1/ 15q24.3	A*24:02	1–2 logs higher in heme.	rs1138357	DYLQ[Y/C**]** VLQI	Y/Y = 6.7 Y/C = 39.5 C/C = 53.5	2.8 (Y) <1 (C)	5.2 (Y) <1 (C)
ACC-6 (46)	HMSD/ 18q21.3	B*4402 B*4403	Leukemia. Not normal hematopoietic	rs9945924	MEIFIEVFSHF	V/V = 10 V/wt = 23 wt/wt = 66.7	2.3	5.9
HA-2 (47)	MYO1G/ 7p13	A*02:01	1–2 logs higher in heme	rs61739531	YIGEVLVS[V**/M]**	V/V = 57 V/M = 38 MM = 6	1.8	2.5
HA-1/B60 (48)	HMHA1/ 19p13.3	B*40:01	1–2 logs higher in heme	rs1801284	KECVL[H**/R**] DDL	H/H = 13 H/R = 46 R/R = 42	1.4	2
LB-ITGB2-1 (25)	ITGB2/ 21q22.3 (transcript variant)	B*15:01	1–2 log higher in heme	rs760462	GQAGFFPSPF	+/+- = 5 +/– = 31 –/– = 63	1	2

*Minor H antigens were selected for inclusion in the table based on (a) having a predominantly hematopoietic gene expression pattern; (b) there being published functional T cell data that clearly demonstrates recognition of hematopoietic cells that endogenously present the minor H antigen(not only peptide-pulsed target cell recognition or tetramer binding); and (c) a reasonably common HLA-restricting allele and a balanced minor H antigen genotype/phenotype distribution such that >1% of the HCT recipients would have the correct HLA and minor H antigen genotype and a donor with the alternative minor H antigen allele*.

There is direct evidence for the anti-leukemic activity of minor H antigen-specific T cells. In humans, donor-derived CD4^+^ and CD8^+^ T cells that have been activated and expanded *in vivo* following recognition of minor H antigens on recipient cells can be isolated and grown *in vitro* and evaluated for anti-leukemic activity (38). Additionally, minor H antigen-specific T cells can be generated by primary *in vitro* stimulation (53). Minor H antigen-specific CD8^+^ T cell clones can inhibit acute myelogenous leukemia (AML) colony growth and lyse primary AML and acute lymphoblastic leukemia (ALL) cells *in vitro* (38, 53–55). Furthermore, minor H antigen-targeting T cells prevent the engraftment of AML in immunodeficient murine models, supporting the hypothesis that early leukemic progenitors are targets of these cells (56).

*In vivo* anti-leukemic efficacy of minor H antigen-specific T cells has also been demonstrated in murine models of HCT and GVL. Perreault and colleagues demonstrated that adoptive transfer of T cells specific for a single immunodominant murine minor H antigen (B6dom1, also known as H7^a^) can eradicate leukemia and has anti-cancer activity in solid tumor models (57–60). Shlomchik and colleagues demonstrated antigen-specific memory T cell (T_M_)-mediated GVL against chronic phase and blast crisis chronic myeloid leukemia (CML) when they transferred CD8^+^ T_M_ from murine donors vaccinated against the H60 minor H antigen (61). In both the Perreault and Shlomchik studies, little to no GVHD was observed when the transferred T cells were specific for a single minor H antigen, even if expression of the minor H antigen was not restricted to the hematopoietic system. However, the anti-tumor efficacy was improved if the minor H antigen was not ubiquitously expressed (57, 59–61). Better efficacy of T cells specific for minor H antigens with hematopoietic-restricted vs. ubiquitous expression can be explained by less activation-induced cell death and T cell exhaustion, and better expansion of T cells targeting hematopoietic-restricted minor H antigens (61).

Focusing the T cell response on a limited number of minor H antigens may favor GVL over GVHD. In mice, leukemia was eradicated following adoptive transfer of CD8^+^ T cells specific for a single broadly-expressed minor H antigen (B6dom/H7a), without the development of GVHD. However, GVHD occurred if B6dom-specific T cells from vaccinated donors were delivered with naïve T (T_N_) cells specific for other minor H antigens (57). Earlier experiments by Korngold and colleagues, using numerous combinations of congenic mouse strains, also did not reveal GVHD in any experiment where donors and recipients were incompatible for single minor H antigens (62). Research by the Falkenburg group using human cells also demonstrated that the magnitude and diversity of the immune response influences the balance between GVHD and GVL (51). They characterized alloreactive CD8^+^ T cell responses in recipients of T cell-depleted (TCD) HLA-matched HCT who achieved a clinical complete response and/or full donor chimerism after donor lymphocyte infusion (DLI). Minor H antigen-specific T cell frequency and diversity was lower in patients with who cleared residual leukemia but did not develop GVHD (i.e., selective GVL) compared to those who developed GVHD and although, patients who developed selective GVL had predominantly hematopoietic-restricted minor H antigen-specific T cells, some did also have T cells that recognized more broadly expressed minor H antigens.

Together, these studies imply that minor H antigens targeted with T cells to augment GVL may not need to be absolutely hematopoietic-restricted from a safety perspective, particularly if the T cell infusion occurs beyond the pro-inflammatory period immediately post-HCT and if a limited number of minor H antigens are targeted. However, targeting minor H antigens that are predominantly expressed on hematopoietic tissue may still be safer, and may be more effective as T cells specific for broadly-expressed antigens tend to become exhausted and dysfunctional (60, 61).

## Minor H Antigen Discovery

A major challenge for developing minor H antigen-targeting, GVL-augmenting strategies is to create therapeutics for *all* patients who may benefit from them, which will require the identification of multiple minor H antigens presented by various HLA types. Techniques used for minor H antigen discovery have been reviewed and include forward and reverse immunology approaches. Forward immunology approaches involve several combinations of different component methods of isolation of activated T cells from HCT recipients, primary *in vitro* stimulation of normal donor T cells, haplotype mapped (HapMap) cell line screening, genetic linkage analysis, genome-wide association studies and cDNA library screening. Combinations of *in silico* analysis, mass spectrometry, HLA/multimer and functional T cell screening have been used in reverse immunology approaches (63, 64).

As there are numerous non-synonymous SNPs (ns-SNPs) with a variant allele frequency between 0.1 and 0.9 across the human genome and a significant minority are encoded by genes that are predominantly expressed in hematopoietic cells, it is anticipated that there remain many minor H antigens that will be suitable targets for therapeutic T cell yet to be discovered. An *in silico* analysis was performed by Lansford et al. to predict minor H antigens. Analysis of recurrent SNPs among 101 HLA-matched HCT recipient donor pairs resulted in the identification of 102 peptides with desirable properties for public, leukemia-associated minor H antigens, specifically with: (a) predicted high binding affinity to a common HLA molecule; (b) RNA expression in AML, but not in GVHD target organs; and (c) optimal allele frequencies to give rise to common minor H antigen mismatches and therefore feasible T cell targeting (52). A proportion of these candidate minor H antigens would be expected to be naturally processed and presented on HLA molecules in leukemic cells, and to elicit a T cell response.

In a second example of the potential for additional minor H antigen discovery, Granados et al. focused on identifying HLA-A^*^02:01 or -B^*^44:03-restricted polymorphic peptides using a mass spectrometry-based proteogenomic approach and cells from 13 volunteer donors and found thousands of candidate minor H antigens (50). Of the nearly 6,773 candidate minor H antigens generated by ns-SNPs, the authors identified 100 relatively common candidates with a minor allele frequency of >0.05, including a set of 39 putative minor H antigens with RNA expression at least two times higher in bone marrow cells than in skin cells. Two of the 39 were tested and induced a T cell response *in vitro*. A proportion of the 39 candidate minor H antigens with predominantly hematopoietic expression would be expected to be both adequately hematopoietically-restricted for therapeutic targeting and immunogenic to have therapeutic utility.

## Examples of HLA Class I Minor H Antigens With Potential for Therapeutic Targeting

### HA-1

HA-1 is the most comprehensively investigated human minor H antigen and is selectively expressed on normal and malignant hematopoietic cells, including AML, myelodysplastic syndromes (MDS), B lineage ALL, and T lineage ALL. The HA-1 peptide epitope is presented on the cell surface in association with HLA-A^*^02:01 and is recognized by HA-1-specific T cells.

The HA-1^H^ peptide (VL**H**DDLLEA) is encoded by a nucleotide sequence spanning a single nucleotide polymorphism (RS_1801284) within the *HMHA1* gene (28). Individuals with a rs1801284 A/A or A/G genotype express the histidine variant (HA-1^H^, also referred to as HA-1) and are considered HA-1 “positive.” Conversely, HA-1 “negative” people with the G/G genotype express only the arginine allelic variant (HA-1^R^, VL**R**DDLLEA), and may have T cells in their repertoire that can respond to HA-1^H^. Both HA-1^H^ and HA-1^R^ undergo similar intracellular processing from the HMHA1 protein, with appropriate proteasomal cleavage and transporter associated with antigen processing (TAP) (29, 30). However, the presence of an arginine at position three of the peptide reduces the affinity of VL**R**DDLLEA binding to HLA-A^*^02:01, relative to VL**H**DDLLEA (29, 30). HA-1^H^-specific T cells do not recognize VL**R**DDLLEA at peptide concentrations that are naturally presented on cells. Therefore, HA-1-specific T cells can be employed following HCT to selectively target residual leukemic cells from a HA-1 positive patient, without damaging normal hematopoietic cells of HA-1 negative donor origin.

Approximately 50% of the population presents HLA-A^*^02:01, and HA-1 allelic variants are phenotypically balanced in the population (rs1801284 A/A 16%, A/G 36%, G/G 48%; HA-1 positive 52%, HA-1 negative 48%). This means that ~10–15% of the HCT population will express both HA-1^H^ and the HLA-A^*^02:01-restricting allele and also have a suitably mismatched HA-1 negative or HLA-A^*^02:01 negative donor, making HA-1 a relatively feasible minor H antigen target for T cell immunotherapy.

Multiple publications have documented that *HMHA1* gene expression is very low to absent in non-hematopoietic cells (65–67), and that HA-1 genotypically-positive hematopoietic cells but not non-hematopoietic cells are recognized by HA-1 -specific T cells *in vitro* (37). Furthermore, HA-1-specific T cells induce little or no tissue damage when co-cultured with HA-1 positive skin biopsy specimens (68). Following HCT from an HA-1 negative donor to an HLA-A^*^02:01 HA-1 positive recipient, HA-1-specific T cells can be identified in approximately one-third of patients (69). One study reported a temporal relationship between the presence of HA-1-specific T cells and GHVD early post-HCT (70), but HA-1-specific T cells have been identified in patients without GVHD following DLI therapy (51, 71–73). Additionally, associations between HA-1 donor-recipient genotypic disparity and GVHD have been observed in some (74–77) but not all (78–81) HCT studies. One explanation for this apparent inconsistency is that patient hematopoietic cells remain in the tissues for several months before being replaced by donor-derived cells (72). Thus, HA-1-specific T cells generated by *in vivo* priming following HCT may contribute to GVHD early post-HCT but are less likely to do so in the context of DLI or delayed HA-1 targeted T cell immunotherapy.

Circumstantial evidence suggests that HA-1 can serve as a therapeutic target in the context of HCT and unmanipulated DLI. Specifically, there have been several reports in which patients with hematological malignancies responded DLI to treat recurrent disease following HCT and coincident emergence of HA-1-specific T cells *in vivo* was documented using peptide/HLA tetramer analysis and/or isolation of the HA-1-specific T cells from the peripheral blood (29, 51, 71–73). In some of these reports HA-1-specific T cell clones isolated from the patients were further evaluated and demonstrated specific killing of HLA-A^*^02 positive^+^ HA-1^H^ -pulsed target cells and primary leukemic cells (71–73). There have also been reports of HA-1 positive patients with multiple myeloma who were treated with dendritic cell vaccines loaded with minor H antigen peptides and adjuvant followed by DLI. Several patients developed a detectable HA-1-specific T cell responses, without adverse effects and two achieved disease control for 6–7 months (82, 83). Together, these observations suggest HA-1 will be a safe and effective target for T cell immunotherapy. HA-1-directed T cell immunotherapy is currently in development, as discussed below.

### BCL2A1/ACC-1 and ACC-2

*BCL2A1* is a member of the Bcl-2 family of anti-apoptotic genes. Two minor H antigens, ACC-1^Y^ and ACC-2^D^, result from distinct nucleotide polymorphisms in the *BCL2A1* gene and are presented by HLA-A^*^24:02 and HLA-B^*^44:03, respectively (44). *BCL2A1* is frequently highly expressed in hematologic malignancies and may contribute to the malignant phenotype, making ACC-1 and ACC-2 attractive targets for T cell immunotherapy (84, 85).

The polymorphisms, rs1138357 and rs3826007, lead to single amino acid substitutions in exon 1 of *BCL2A1*, creating the immunogenic HLA-A^*^24:02-restricted ACC-1^Y^ [DYLQ**Y**VLQI, rs1138357 AA (6%) or AG (39%)] and B^*^44:03-restricted ACC-2^D^ [KEFED**D**IINW, rs3826007 AA (4%) or AG (50%)] (44). ACC-1-specific T cells can be generated from HLA-A^*^24:02 positive ACC-1 “homozygous negative” donors (rs1138357, GG encoding DYLQ**C**VLQI) and distinguish the single amino acid difference (tyrosine vs. cysteine in ACC-1). Similarly, ACC-2-specific T cells from homozygous negative donors (rs3826007, GG encoding KEFED**G**IINW) can distinguish the aspartic acid from the glycine.

ACC-1 and ACC-2 are feasible targets for T cell immunotherapy. Based on the prevalence of the HLA-A^*^24:02 and HLA-B^*^44:03 restricting alleles and the frequency of distribution of immunogenic and non-immunogenic variants of ACC-1 and ACC-2, the calculated estimate of finding a polymorphism-discrepant, matched related and unrelated donor is 2.8 and 5.2% for ACC-1, and 3.6 and 6.7% for ACC-2, respectively (86).

There has been controversy regarding how selectively *BCL2A1* is expressed in hematopoietic cells. Gene expression analysis by Northern blot (44), quantitative polymerase chain reaction (67), and database microarray (http://biogps.gnf.org) suggests hematopoietic-restricted expression. Although the Goulmy group showed that *BCL2A1* expression could be upregulated in non-hematopoietic cells (mesenchymal stromal cells) by simultaneous exposure to interferon gamma (IFNγ) and tumor necrosis factor alpha (TNFα), but not to IFNγ alone (87), Akatsuka's research group subsequently demonstrated comparable up-regulation of *BCL2A1* and *HMHA1* after administration of IFNγ and TNFα (67). In any case, the clinical relevance of upregulation of minor H antigen-encoding genes after exposure to high doses of cytokines *in vitro* has not been established. ACC-1^Y^ and ACC-2^D^ genotypically positive non-hematopoietic cells are not recognized in cellular assays without exogenous cytokines (44, 67, 87). Moreover, an analysis of HCT outcomes in 320 patients expressing HLA-A^*^24:02 did not show an increase in GVHD in recipients with an immunogenic *BCL2A1* allele and an antigen negative donor (88). Together these data suggest that ACC-1^Y^ is likely to be a safe target.

ACC-1- and ACC-2-specific T cells lysed primary leukemic cells *in vitro* after isolation from HCT recipients (44). In a subsequent study, direct tetramer analysis was used to identify ACC-1-specific T cells in the bone marrow and peripheral blood in a patient who received an HLA-matched, ACC-1-disparate HCT for CML 14 months earlier (89). The bone marrow ACC-1-specific T cells proliferated in response to ACC-1 peptide stimulation and demonstrated cytotoxicity against a cell line endogenously presenting ACC-1, demonstrating the presence of functional ACC-1 specific T cells that may contribute to protection against post-HCT relapse. Gene expression data suggests that BCL2A1 is not expressed at high levels in all leukemia cells. Although high-level expression is not necessarily required to render leukemia susceptible to lysis by high-avidity minor H antigen-specific T cells, functional assays demonstrating lysis of numerous ACC-1 and ACC-1 genetically positive leukemia will be necessary before ACC-1 and 2 directed T cell immunotherapy is advanced to the clinic.

### P2X5/P2RX5/LRH-1

The *P2RX5* gene is a member of the P2X purinergic ATP-gated non-selective cation channel protein group and has been recognized as promoting cancer growth and aggression (90–93). A frameshift polymorphism (rs3215407) in *P2RX5* leads to major differences in the *P2RX5*-transcribed sequence, including the immunogenic HLA-B^*^07:02-restricted LRH-1 minor H antigen TPNQRQNVC (22). On the basis of the frequency of the HLA-B^*^07:02 restricting allele and 46% prevalence of the homozygous cytosine deletion, the calculated estimate of finding discrepant matched related and unrelated donors is 4.9 and 7.5%, respectively (86). If HCT donors have the rs3215407 polymorphic sequence with a homozygous deletion of a cytosine at position 732, they may generate T cells that recognize the TPNQRQNVC minor H antigen generated in individuals without the cytosine deletion.

The *P2RX5* gene is expressed in normal lymphocytes, B and T lineage ALL, a range of lymphoma and multiple myeloma cases, the CD34^+^ fractions of CML and AML, and possibly at low levels in brain and skeletal muscle, but there is minimal expression in GVHD target tissues (intestine, liver, lung, skin) (22). LRH-1 genotypically-positive fibroblasts, a representative non-hematopoietic tissue, are not recognized in cellular assays (22).

LRH-1-specific T cells kill ALL CD34^+^, AML CD34^+^, CML CD34^+^, and multiple myeloma CD138^+^ cells *in vitro* (22, 94, 95). Moreover, LRH-1-specific T cells have been detected in the peripheral blood of patients responding to DLI (22, 95, 96). Dolstra's group studied seven HLA-B^*^07:02^+^ LRH-1 positive patients who received HCT and DLI from a HLA-B^*^07:02 positive LRH-1 negative donor, and detected LRH-1-specific T responses coinciding with declines in detectable leukemia in three of the seven patients, including two patients with CML and one with AML (22, 95). Gene expression data indicates that LRH-1 is not expressed at high levels in all leukemia blasts or progenitors, although progenitors with relatively low levels of LRH-1 expression can be inhibited by LRH-1-specific T cells in functional assays (22, 95). Further functional assays demonstrating activity of LRH-1-specific T cells against multiple LRH-1 genetically positive leukemia cell targets *in vitro*, and ideally in patient-derived murine xenograft models, are required before translation of LRH-1-specific T cell immunotherapy to the clinic.

## HLA Class II Minor H Antigens

The primary focus of minor H antigen discovery has been on HLA class I-restricted minor H antigens as targets for CD8^+^ T cells. However, class II-restricted minor H antigens are also of interest particularly given that HLA class II molecules are generally expressed at relatively low levels on non-hematopoietic cells under non-inflammatory conditions. As such, class II-restricted minor H antigens may be more likely to induce a selective GVL response even if the gene encoding the minor H antigen is relatively broadly expressed. In a recent study of CD4^+^ enriched DLI from HLA-identical sibling donors, GVL reactivity without GVHD was associated with CD4^+^ T cells targeting HLA class II-restricted minor H antigens, some of which were associated with genes expressed in non-hematopoietic cells (97). However, HLA class II gene expression is often downregulated on leukemic cells after HCT (98, 99), which implies that while class II-restricted minor H antigen-specific T cells may make a major contribution to GVL after HCT and drive the HLA class II downregulation, class II-restricted minor H antigens may not be optimal targets for T cell immunotherapy to treat post-HCT relapse.

## T Cell Immunotherapy Targeting Minor H Antigens (Figure 4)

Minor H antigens have several advantages as targets for therapeutic T cells aimed to prevent or manage relapse. First, minor H antigens arise from germline variants and are therefore likely to be expressed, at least initially, in all leukemia cells in an individual, unlike neoantigens that are often subclonal, permitting escape from neoantigen-specific T cells. Second, minor H antigens are foreign to donor T cells, like neoantigens and unlike overexpressed non-polymorphic leukemia-associated antigens. High-affinity minor H antigen-specific TCRs can be relatively readily found in the repertoire of normal donors and exploited as therapeutics (100). Lastly, as described above, minor H antigen expression is inherently specific to HCT-recipient cells, and therefore specific to recipient leukemia after myeloablative HCT. This inherent specificity avoids many of the challenges for chimeric antigen receptor T cells (CAR-T) that target cell surface antigens that are shared with normal recipient or donor hematopoietic cells, leading to problems with prolonged marrow aplasia in the case of AML CAR-T, for example.

**Figure 4:**
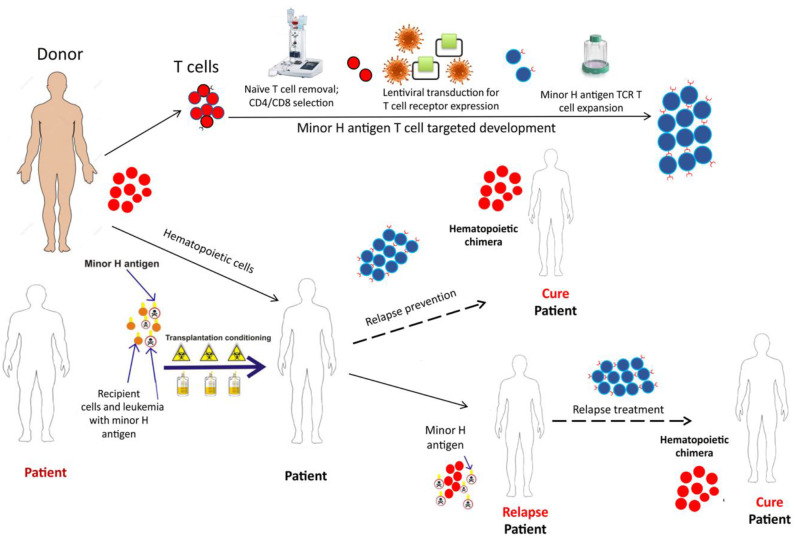
The schematic depicts the development and potential therapeutic use of donor derived minor H antigen targeting T cell therapy. A patient, with the hematopoietically-restricted minor H antigen target, would undergo allogeneic HCT. The donor, lacking the minor H target, would donate hematopoietic cells for the HCT graft and T cells for the immunotherapy product. The T cells would undergo depletion of the naïve T cell fraction (to reduce GVHD risk) and then undergo modification using a lentiviral construct containing a potent TCR targeting the minor H antigen of interest. Following this modification, the cell product would be expanded to develop a therapeutically active cell dose which could be infused soon after the HCT as a relapse prevention strategy or following relapse detection to treat post-HCT relapse.

Immunotherapy employing T cells genetically modified with transgenic TCR alpha and beta chains is a promising strategy for treating hematologic malignancies and solid tumors (101, 102). Genetic TCR transfer into a selected T cell subset facilitates relatively rapid production of a T cell immunotherapy product with high potential for expansion, function and persistence after infusion into the patient. We developed T cell immunotherapy employing donor T_M_ cells transduced with a lentiviral vector encoding a TCR specific for HA-1 and are currently evaluating this novel therapeutic in a phase I clinical trial for the treatment of post-HCT MRD or relapse (NCT03326921) (100). The cell product incorporates multiple elements designed to optimize efficacy and safety: (1) a high-avidity HA-1 specific TCR with potent anti-leukemic activity; (2) a CD8^+^ co-receptor to promote function of the class I-restricted TCR in CD4^+^ T cells; (3) an inducible caspase 9 safety switch that can be triggered by the dimerizer AP1903/rimiducid in the event of unexpected side effects; (4) a CD34-CD20 epitope to facilitate selection and tracking of the engineered T cells; and (5) predominantly central memory T cells to promote persistence after infusion and to avoid infusing GVHD-inducing T_N_ cells (100, 103, 104). This is the currently the only minor H antigen targeting clinical trial enrolling pediatric patients that we are aware of. Another HA-1 TCR T cell immunotherapy trial (MDG1021) will open in Europe this year, employing a HA-1 TCR T cell product developed in Leiden (105, 106).

Of note, while current clinical trials targeting minor H antigens are evaluating the safety profile of minor H antigen-specific T cells as treatment for post-HCT leukemia recurrence, a longer-term goal is to deliver hematopoietic-restricted minor H antigen-specific T cells soon after the HCT graft to augment GVL and prevent relapse.

## Graft Engineering to Augment the T Cell Response to Selected Minor H Antigens

The term “graft engineering” refers to manipulation of the composition of cells collected from an allogenic HCT donor prior to infusion into the patient. To date, graft engineering has primarily involved selections and/or depletions of particular cell subsets for the purpose of mitigating the risk of GVHD. However, more complex manipulations can be considered with emerging technology, including enrichment for rare antigen-specific T cells, genetic modification of cells with CAR-T or TCR-T, and/or knockdown of genes in order to protect certain cells or augment their function. Here we will discuss recent progress in graft engineering to create an improved platform for delivery of antigen-specific T cells, and to develop strategies for enhanced delivery of leukemia-associated minor H antigen-specific T cells.

Minimizing the risk of serious GVHD is a critical first step to enabling the effective delivery and function of hematopoietic-restricted, minor H antigen-specific T cells, since the management of GVHD requires pharmacologic immunosuppression that is not conducive to T cell expansion and function. Non-selective removal of T cells from the donor graft can be achieved through pre-infusion physical depletion of T cells or enrichment of CD34^+^ stem cells using immunomagnetic beads, or by *in vivo* depletion using T cell antibody-directed therapy. Non-selective T cell depletion strategies have led to reduced GVHD rates but also increased infection risk due to prolonged immune reconstitution (107–111). Selective T cell depletion strategies, including CD45RA^+^ T_N_ depletion and αβ TCR T cell depletion, are aimed at reducing GVHD but retaining lymphocytes with activity against pathogens and malignant cells and are being evaluated in children and adults.

### T_N_-Depletion

Mature αβ TCR T cells can be divided into categories based on differentiation: naïve, T memory stem cells, central memory, effector memory, and effectors (112). Naïve T cells (T_N_) which are antigen inexperienced, include cells that can react to minor H antigens expressed on epithelial tissues following recipient infusion, resulting in GVHD. Murine modeling using MHC matched and mismatched mice have demonstrated that antigen experienced T_M_ cells cause less or no GVHD compared to T_N_ (113–121). They do, however, show appropriate antigen-specific T cell responses and beneficial GVL (114, 120). Additionally, *in vitro* studies found that human minor H antigen-specific T cells were more prevalent among T_N_ than T_M_ cells (53). Bleakley et al. developed a technique to engineer CD34^+^ cell-enriched, T_N_-depleted peripheral blood stem cells (PBSC) for HCT (122). Initial published experience has demonstrated remarkably low levels of chronic GVHD without increases in relapse or infection rates following HCT of T_N_-depleted HLA-matched related donor PBSC in patients with leukemia (104). Additional unpublished data shows comparable results in a larger cohort of pediatric and adult recipients of T_N_-depleted PBSC from HLA-matched related and unrelated donors. This approach is currently being further studied in randomized prospective trials comparing the T_N_-depleted PBSC to conventional bone marrow stem cells for pediatric patients (NCT03779854) and to alternative strategies for GVHD reduction in adults (NCT03970096).

### αβ TCR T Cell Depletion (αβ-TCD)

HCT strategies that deplete the donor stem cell graft of all αβ TCR T cells to remove alloreactive T cells and CD19 B cells to avoid EBV post-transplant lymphoproliferative disorder, while retaining γδ TCR T cells and NK cells, are also being investigated. HCT with αβ-TCD grafts shows promise as a platform to reduce GVHD, especially in pediatric patients (123–128). γδ TCR T cells and NK cells both have activity against pathogens and malignant cells, so their retention in the graft may protect patients against infection and relapse, respectively (128–134). In long-term analysis of 98 pediatric patients with leukemia, the outcomes of patients who received haploidentical αβ-TCD grafts were encouraging with 3-year DFS, relapse, severe acute and extensive chronic GVHD rates of 62, 29, 0, and 1%, respectively (126, 127). Moreover, in a large retrospective analysis comparing outcomes of unmanipulated HLA matched or mismatched unrelated donor HCT (MUD; MMURD) with haploidentical αβ-TCD HCT, 5-year chronic GVHD free, relapse free survival (GRFS) was superior in the haploidentical αβ-TCD compared to the MMURD (135).

Assuming continued success in reducing GVHD by graft engineering, the next challenge is to augment HCT grafts for enhanced anti-leukemic activity, including but not limited to enriching for hematopoietic-restricted minor H antigen-specific T cells. The primary aim of this approach would be to overcome quantitative deficiencies in minor H antigen-specific T cells in the donor HCT graft. Minor H antigen-specific T cells will be numerically deficient in grafts that have been depleted of T_N_ or of all αβ TCR T cells. Additionally, minor H antigen-specific T cells are rare in the donor T cell repertoire even in the absence of any T cell depletion (53) and may not consistently adequately expand, migrate to the bone marrow, and persist with durable anti-leukemic activity after HCT. Antigen-specific T cells are effective at controlling and eliminating cancer cells only when the T cells are present in sufficient numbers relative to the cancer cells. Therefore, particularly for patients with residual disease at the time of HCT, increasing the number of hematopoietic-restricted minor H antigen-specific T cells delivered with the graft should facilitate the GVL effect. Delayed sequential infusion of antigen-specific T cells after infusion of the HCT graft (i.e., a “split graft”) may also be considered to allow the pro-inflammatory state resulting from chemo/radiotherapy conditioning to abate and the recipient tissue-resident normal hematopoietic cells to be largely replaced by cells of donor origin, in order to mitigate the risk of inducing GVHD. Delayed sequential T cell infusions should also circumvent the antigen-induced T cell death that may occur when hematopoietic-restricted minor H antigen-specific T cells encounter residual recipient hematopoietic cells immediately post-HCT, improving persistence. Multiple infusions of cells will also avoid the functional limitation of the donor T cells that results from progressive expression of inhibitory molecules over time (99, 136).

Current hurdles to augmenting HCT grafts for hematopoietic-restricted minor H antigen-specific T cells include the relatively limited number of suitable target minor H antigens that have been discovered and characterized (Table 1) and technical issues largely related to inadequate options for clinical-grade sorting of rare cells. Streptamer technology is being evaluated for isolating antigen-specific T cells (137–140). The technology is based on the direct labeling of CD8^+^ T cells with HLA I-Streptamers composed of peptide-loaded HLA I-Strep-tag fusion proteins reversibly multimerized on magnetically labeled Strep-tactin. After separation, the HLA I-Streptamers can be dissociated from the positively selected cells by the addition of D-Biotin, allowing for rapid enrichment of unlabeled antigen-specific T cells under GMP conditions. However, in a clinical trial of Streptamer-enriched multi-antigen-specific T cells to prevent complications early after T cell-depleted HCT, neither tumor associated antigen or minor H antigen-specific T cells could be confirmed in the majority of antigen-specific T cell products or after HCT, although EBV and CMV-specific T cells were readily detected in the products and sometimes after HCT (140). The greater success in isolating virus-specific T cells compared to minor H antigen- or other tumor-associated antigen-specific T cells, may be due to the relatively high frequencies of virus-specific T cells in the repertoire of normal viral antigen-experienced donors.

Next-generation high-speed cell sorting technology may solve the challenge of isolating very rare cells from donor cell collections. For example, a novel cell sorting technology called OrcaSort™ is being developed by OrcaBio. OrcaSort™ uses fluorescent markers for identification of target cells and high-speed laser pulses to sort cells in fully-enclosed, sterile, disposable cassettes (141). The technology will first be evaluated in clinical trials that require sorting of multiple cell populations for GVHD reduction (NCT03802695, NCT01660607) but could also be adapted to positive selection of antigen-specific T cells.

An alternative strategy for enriching selected minor H antigen-specific T cells in donor cell collections is to first vaccinate the HCT donor against minor H antigens to generate a memory T cell response and increase the frequency of the minor H antigen-specific T cells (61). Shlomchik et al. demonstrated that donor vaccination with recipient minor H antigens, and subsequent transplantation of donor T memory T cells, transferred leukemia- and pathogen-specific immunity in murine bone marrow transplantation (BMT) recipients (61). The transferred memory T cells expanded after BMT and augmented GVL. This approach could be translated to humans by vaccinating donors months before HCT or intended post-HCT infusion and then specifically selecting the minor H antigen T cells using a immunomagnetic bead-based technique, such as Streptamer selection (137–140) or the Miltenyi Biotec CliniMACs Cytokine Capture System (142, 143). Alternatively, after donor vaccination with hematopoietic-restricted minor H antigens, donor T cells could be collected and depleted of naïve T cells to avoid GVHD (104), and the minor H antigen-specific T cell enriched memory T cells could be delivered with or following the stem cell graft. Minor H antigen vaccination of donors is likely to be safe, given that clinical trials of vaccination of HCT recipients against minor H antigens have been completed without excess toxicity (82, 83). Donor vaccination could also be employed to improve the efficacy of DLI to prevent or treat post-HCT relapse by producing a product enriched for particular minor H antigen-specific T_M_ cells. The DLI product could be further engineered by depletion of T_N_.

## Hematopoietic Cell Transplantation for Pediatric Leukemia in the Current Era and Future

Allogeneic HCT is the current standard of care for pediatric patients with very high-risk hematologic malignancies (1, 2). The overall success of HCT as a treatment for pediatric leukemia has improved, with reduced non-relapse mortality rates (144) and leukemia-free survival rates in the range of 60–80% (145, 146) in the current era. However, conditioning regimens and GVHD have long lasting adverse effects, particularly for the pediatric population who undergo significant neurologic and physical development. Long-term effects of HCT, particularly of conditioning and especially related to TBI, include growth disturbance, hormone deficiencies, cataracts, seizures, cerebrovascular events, dyslipidemia, and secondary malignancies (12–16). GVHD can also lead to long-lasting morbidity involving multiple organs, most commonly the skin, gut, and liver, but also lungs, mouth, eyes and joints in the chronic setting (15, 17).

The mortality and morbidity of HCT may be improved by graft engineering and adjunctive T cell immunotherapy to reduce GVHD and to augment GVL. Because T cell immunotherapy has the potential to prevent relapse, it may permit de-escalation of conditioning intensity, which will be critically important for the youngest HCT recipients in whom myeloablative HCT can be associated with devastating neurodevelopmental complications (13). In infants, leukemia is often refractory to chemotherapy and relapse occurs frequently (147). HCT is indicated for infants with ALL in first remission with the highest risk of relapse, and for those who achieve a second remission after relapse. Unfortunately, the youngest infants with ALL generally have the highest risk of relapse with chemotherapy alone, but also the greatest risk of late effects of HCT conditioning. Because of this combination of high risk from both disease and complications of therapy, there is an urgent need to develop reduced toxicity HCT strategies for infants, supplemented by add-back of minor H antigen-specific T cells and/or post-HCT T cell immunotherapy to prevent relapse.

The presence of detectable disease at the time of HCT is consistently and strongly associated with an increased risk of relapse post-HCT (6, 7). As such, other important recent developments that enable reduction of conditioning intensity include sensitive techniques for detecting measurable residual disease (MRD) and therapies for reducing MRD prior to HCT. These advances are particularly important when one contemplates HCT strategies that rely on the T cell-dependent GVL effect, which is relatively delayed compared to the immediate anti-leukemic effect of intensive conditioning. MRD detection has moved from morphologic evaluation to the use of flow cytometry or PCR, which have sensitivities of 0.1% for AML and 0.01% for ALL (148, 149). Technologies to detect even lower levels of disease have been developed and the clinical implications are being studied. Next-generation sequencing (NGS) measuring immunoglobulin heavy chain (variable, diversity and joining) have been developed for ALL; patients who are NGS negative prior to HCT have been shown to have a reduced risk of relapse (150). NGS is also currently being evaluated for AML (151, 152) and may prove useful for risk stratification and perhaps monitoring, but is likely to be complex (153). Highly sensitive MRD evaluation in the pre-HCT period may be used to guide which pediatric patients can undergo HCT with reduced intensity/toxicity conditioning without an excessive risk of relapse, and which patients do have a high risk of relapse and may benefit from the addition of novel relapse prophylaxis, such as minor H antigen-specific T cells. In cases where MRD is detected pre-HCT, new targeted strategies, particularly CAR-T cell immunotherapy and bispecific T cell engagers, can also produce deep MRD negative remissions prior to HCT and thereby may improve post-HCT prognosis (154–158).

Significant success has been achieved using CAR-T cell immunotherapy targeting lineage-specific antigens to treat pediatric and adult patients with acute leukemia. This is highlighted by the efficacy of some CAR-T cell products targeting CD19, an antigen expressed on normal and leukemic B cells in some acute leukemias and lymphomas (154, 156). Patients who have received effective CD19 CAR-T cell therapy develop B cell aplasia and hypogammaglobulinemia as a result. Given hypogammaglobulinemia can be supported with intravenous immunoglobulin administration, and patients do not have significant infectious complications (159), B cell aplasia is not a major barrier for CD19 CAR-T cell immunotherapy. Targeting myeloid-lineage antigens, such as CD33 or CD123, is likely to be more problematic due to the risk of marrow suppression or aplasia placing patients at risk for severe associated complications including infection (160, 161). Minor H antigen-targeted therapies are not suitable for use in patients who have not received allogeneic HCT and therefore have a recipient hematopoietic system. However, following HCT the presence of a normal donor hematopoietic system lacking the minor H antigen target limits the therapeutic targets to neoplastic cells or residual recipient normal cells that are no longer necessary, and should protect the recipient from marrow suppression, B cell aplasia, and other hematopoietic complications. Additionally, many CAR constructs incorporate non-human components, frequently using murine derived svFc, which can be immunogenic leading to a rejection response, whereas the TCR in minor H antigen-specific T cells and TCR-T cell products are of human origin and are inherently less immunogenic favoring *in vivo* persistence (162). Lastly, although minor H antigen-targeted therapy is restricted to patients with the restricting HLA-allele and appropriate recipient-donor mismatch for the polymorphism, minor H antigen-targeted therapy is not limited to one leukemia subtype, in contrast to most CAR-T cell immunotherapy.

Ultimately, superior survival with reduced morbidity after pediatric HCT could be achieved by a combination of (a) therapies to achieve remission without any detectable disease, potentially including CAR-T cell therapy or other immunotherapy (b) a reduced-intensity/toxicity conditioning regimen, (c) a HCT graft selectively depleted of GVHD-inducing T cells and augmented with T cells specific for a limited number of hematopoietic restricted minor H antigens, and (d) post-HCT minor H antigen-targeted T cell immunotherapy. Although this vision has not yet been reached, and minor H antigen-targeted T cell therapies are currently in early phase clinical trials without proven benefit for pediatric or other patients, steady progress is being made in the fields of antigen discovery, graft engineering and genetically-modified T cell therapy. We anticipate that these advances will continue and come together to benefit children with very high-risk leukemia.

## Conclusions

Donor-derived T cell responses to minor H antigens with hematopoietic-restricted expression are a key component of effective GVL. Targeting leukemia-associated minor H antigens with T cells in hematopoietic grafts and/or with post-HCT T cell immunotherapy are potentially low-toxicity, high-efficacy prophylactic and therapeutic strategies for pediatric patients. Pre-clinical studies and clinical trials of T cell immunotherapies to enhance the response to hematopoietic-restricted minor H antigens are underway. These advancing technologies should enable a reduction in HCT conditioning intensity without sacrificing protection from post-HCT relapse, thereby mitigating dangerous late effects of HCT for pediatric patients with leukemia.

## Author Contributions

CS, VS, and MB reviewed the literature, wrote, and edited the manuscript.

## Conflict of Interest

MB is a Founder and Scientific Advisory Board member of HighPassBio, a Scientific Advisory Board member of Orca Bio, and has also received compensation from Miltenyi Biotec for presentations at conferences and corporate symposia. The remaining authors declare that the research was conducted in the absence of any commercial or financial relationships that could be construed as a potential conflict of interest.
